# Reducing Measurement Time in Direct Interface Circuits for Resistive Sensor Readout

**DOI:** 10.3390/s20092596

**Published:** 2020-05-02

**Authors:** José A. Hidalgo-López, José A. Sánchez-Durán, Óscar Oballe-Peinado

**Affiliations:** 1 Departamento de Electrónica, Universidad de Málaga, Andalucía Tech, Campus de Teatinos, 29071 Málaga, Spain; jsd@uma.es (J.A.S.-D.); oballe@uma.es (Ó.O.-P.); 2 Instituto de Investigación Biomédica de Málaga (IBIMA), 29010 Málaga, Spain

**Keywords:** direct interface circuits, calibration methods, error analysis, resistive sensor, interface sensor, time-based measurement

## Abstract

Direct Interface Circuits (DICs) carry out resistive sensor readings using a resistance-to-time-to-digital conversion without the need for analog-to-digital converters. The main advantage of this approach is the simplicity involved in designing a DIC, which only requires some additional resistors and a capacitor in order to perform the conversion. The main drawback is the time needed for this conversion, which is given by the sum of up to three capacitor charge times and their associated discharge times. This article presents a modification of the most widely used estimation method in a resistive DIC, which is known as the Two-Point Calibration Method (TPCM), in which a single additional programmable digital device pin in the DIC and one extra measurement in each discharge cycle, made without slowing down the cycle, allow charge times to be reduced more than 20-fold to values around 2 µs. The new method designed to achieve this reduction only penalizes relative errors with a small increase of between 0.2% and 0.3% for most values in the tested resistance range.

## 1. Introduction

Electrical magnitudes of sensors can currently be read using a wide range of circuits, including Direct Interface Circuits (DICs), which comprise several features that make them particularly suitable for multiple applications. A DIC performs a magnitude-to-time-to-digital conversion through a programmable digital device (PDD) and a few additional elements. The first versions of DICs were proposed, almost simultaneously, by Sherman and Webjörn [1,2].

Reading a sensor with a DIC produces a digital output that can be processed directly by the same PDD as used for the measurement, without the need for analog-to-digital conversion. Indeed, only a few simple extra elements are required, such as resistors, capacitors, transistors, or triggers [3]. Such simplicity means that this type of circuit is particularly suited for portable applications, where both the number of components and their consumption are very important. DICs are also highly versatile in terms of the PDDs used, allowing the use of both microcontrollers [4,5,6,7,8,9,10] and field-programmable gate arrays (FPGAs) [11,12,13,14,15,16]. The calculation capabilities of a PDD connected to a DIC allow them to function as smart sensors, pre-processing information from the sensor and thus reducing the workload in subsequent high-level processing and decision stages [17].

Although there are DICs for reading both capacitive and inductive sensors, those for resistive sensors have probably been the most widely used and analyzed in the literature, whether individually [3,7,18,19] or grouped in arrays [11,14]. Several issues need to be considered when designing a resistive DIC, such as accuracy, uncertainty, resolution [6,8,16,20,21,22], and calibration points [23]. Other problems that may influence the performance of these circuits such as response to dynamic signals [24], lead wire resistance [25], power consumption [5], estimation time [13], or problems related to the measurement of low-resistance sensors [15], have also been addressed.

However, problems related to variations in the charge voltage on the capacitor used in a resistive DIC have not received as much attention in the literature. These variations are fundamentally related to two causes. Firstly, there is the interference effect of the power supply in the voltage stored on the capacitor. This effect has only been studied in [26]. This article showed how results can be improved when charging the capacitor through an additional resistor connected to a PDD output and to the capacitor itself. Nevertheless, this solution presents a new problem, namely the increase in charge time due to the presence of this resistor. Secondly, the length of this charge time limits the operation of a resistive DIC in relation to the capacitor’s final charge voltage, since precise measurements can only be achieved if the capacitor’s different charges (as necessary in the resistive sensor reading process) reach the same voltage; this means a high charge time is required, thus minimizing any differences in the initial charge voltages [27] showed that the charge time must be at least five times the time constant formed, firstly, by the sum of the additional resistance and the resistance presented by a PDD output buffer, and, secondly, by the capacitor. Thus, if *T_E_(R_S_)* refers to the total time for estimating the resistance value of a sensor, *R_S_*, this time will increase notably due to this additional resistance, which, even when it has a small value as in [26], will always be much higher than the PDD buffer’s small output resistance value.

This article will set out a new method to offset the effects of variations in the final voltage on the capacitor charge cycles of a resistive DIC. This new method can be used to reduce errors when estimating the resistance value of the sensor due to this reason, or to reduce the capacitor’s charge time, thus decreasing *T_E_(R_S_)*.

The structure of the paper is as follows. Section 2 shows the operating principles of the different types of DICs and their fundamental characteristics. Section 3 presents the new DIC proposal. Section 4 shows the materials and methods used in the evaluation of the new proposal and the experimental results. Finally, the conclusions are presented in Section 5.

## 2. Operating Principle of a DIC

**Figure 1:**
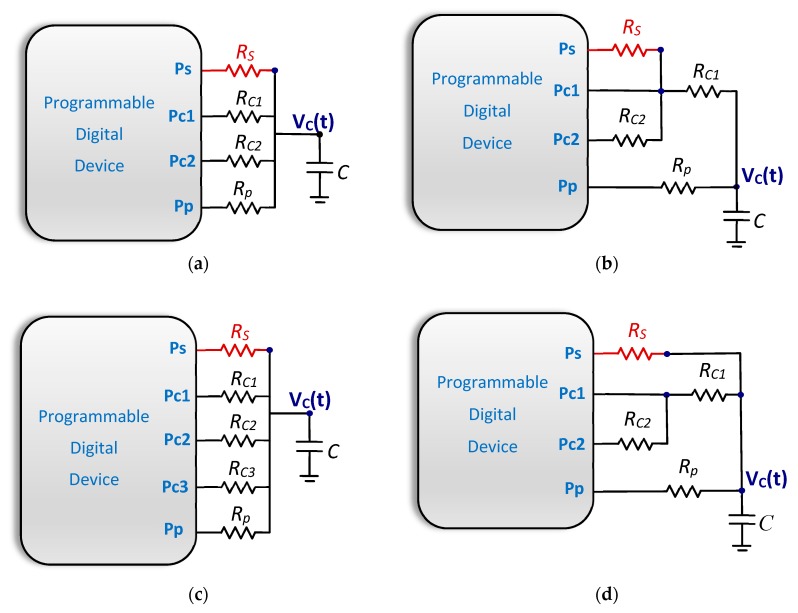
Different types of resistive Direct Interface Circuits (DICs) proposed in the literature (resistor/sensor to be estimated shown in red). (**a**) Two-Point Calibration Method, TPCM. (**b**) Three-Signal Method, TSM. (**c**) DIC for measuring low resistance values. (**d**) DIC designed to reduce quantization error.

The DIC most frequently used in the literature for reading resistive sensors uses the Two-Point Calibration Method, TPCM. This has the structure shown in Figure 1a. The *R_S_* reading process consists of three cycles. In the first part of each cycle, capacitor *C* is charged to a voltage, *V_ch_*, which is ideally equal to the supply voltage, *V_DD_*. This is done by placing a logic 1 output in the PDD’s Pp pin and charging *C* through resistor *R_p_* (added in accordance with the indications of [26]). During the charge process, pins Ps, Pc1, and Pc2 are in a high-impedance state, HZ (i.e., configured as inputs). Then, the discharge of *C* takes place by placing the logic 0 output in one of the PDD’s Ps, Pc1, and Pc2 pins. Only one of these three pins will be configured as logic 0 output during the discharge processes, with the other two and Pp configured in the HZ state. Therefore, one discharge takes place through *R_S_*, and the other two take place through two known calibration resistors, *R_C1_* and *R_C2_*. The voltage of the capacitor, *V_C_(t)*, will evolve according to the following equation during the discharge process through *R_S_*:

(1) $$V_{C}(t)=V_{ch}e^{\frac{-t}{\left(R_{S}+R_{o}\right)C}}$$

where *R_o_* is the output resistance of each pin configured as logic 0 output. The discharge ends when it is detected that the voltage in *C* has dropped below the threshold voltage *V_Th_* of the Pp pin (configured as input). Equation (1) can be used to establish the time taken in this discharge, *T_Rs_*:

(2) $$T_{Rs}=\left(R_{S}+R_{o}\right)C\ln\left(\frac{V_{ch}}{V_{Th}}\right)$$

Discharging through *R_C1_* and *R_C2_* instead of through *R_S_* achieves times *T_Rc1_* and *T_Rc2_*, replacing *R_S_* with *R_C1_* and *R_C2_* in Equation (2). If the noise in *V_DD_* is low and the charge times are long enough for discharges to always start from the same voltage on the capacitor, *V_ch_* ≈ *V_DD_*, then the value of *R_S_* can be expressed straightforwardly in accordance with these times [4]:

(3) $$R_{S}=\frac{T_{Rs}-T_{Rc1}}{T_{Rc2}-T_{Rc1}}\left(R_{C2}-R_{C1}\right)+R_{C1}$$

This is the known equation for finding *R_S_* in the TPCM. The times shown in this equation represent integers of the PDD clock period, *T_CK_*. Therefore, *R_S_* can be found through known resistance values and the three-time measurement, eliminating dependence on *C*, *V_ch_*, and *V_Th_*.

A variant of this resistive DIC, which uses the same passive components (although arranged differently) and a different calibration method, is the Three Signals Method or TSM, as shown in Figure 1b. Three charge–discharge cycles are also carried out in the TSM, although in this case, discharge (duly configuring the pins) takes place through *R_C1_*, *R_C2_* + *R_C1_*, and *R_S_* + *R_C1_*. This method, with the same *V_ch_* restrictions as those indicated for the TPCM, can be used to find *R_S_* from the following expression [4]:

(4) $$R_{S}=\frac{T_{Rs+Rc1}-T_{Rc1}}{T_{Rc2+Rc1}-T_{Rc1}}R_{C2}$$

This expression is easier to evaluate than Equation (4), but the method has more inaccuracies in determining *R_S_* [13].

A second variant of the TPCM, Figure 1c, uses an additional calibration resistor, *R_C3_*, whenever low *R_S_* measurements are needed. This means the circuit requires an additional charge and discharge process, but it allows *R_S_* to be determined with 10 times greater precision if its value is around 10 Ω [15].

Another resistive DIC, with a different arrangement of resistors, is shown in Figure 1d [16]. In this case, three charge and discharge cycles are also carried out. However, two pins detect changes from 0 to 1 during discharge through *R_S_*, namely Ps and Pc1. Since Pc1 will always detect the change before Ps, there is an option to continue discharge from this moment of detection, either through *R_S_* or *R_C1_* + *R_C2_*. This method reduces uncertainty due to quantization in *T_Rs_* when the measurement value is low, which occurs if *R_S_* is also low, thus achieving an improvement in accuracy by determining *R_S_* for these resistance values. However, it comes at the expense of increased complexity of the equations that determine the value of *R_S_* and the need to save two time measurements during the discharge of *R_S_*.

Resistor *R_p_* was introduced in these resistive DICs to reduce noise in the capacitor charge, thanks to the low-pass filter formed by *R_p_* and *C*. However, as mentioned above, this solution has the disadvantage of increasing the time needed to charge *C* and, therefore, the total estimation time, *T_E_(R_S_)*. As indicated above, long charge times are needed since the maximum voltage the capacitor is charged at (*V_ch_*) must be equal in all charge cycles and independent of the initial voltage each charge begins at (*V_C_(0)*), meaning, for example, Equations (3) and (4) remain valid (the same is true for the circuits in Figure 1c,d). This can be seen in the charge equation of a capacitor through the Pp pin:

(5) $$V_{C}(t)=V_{DD}+\left(V_{C}(0)-V_{DD}\right)e^{\frac{-t}{\left(Rp+Ro\right)C}}$$

In this equation, the influence of *V_C_*(0) on the final voltage stored on the capacitor, *V_ch_*, is only negligible if *t* is large.

**Figure 2:**
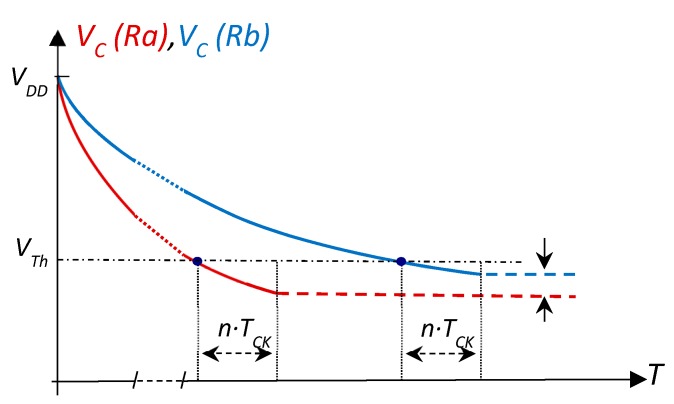
The *C* discharge process through two different resistors, *Ra* and *Rb* (curves *V_C_(Ra)* and *V_C_(Rb)*) ends in different voltages due to the prolongation of the discharge for a certain number of clock cycles, *n·T_CK_*.

However, the initial voltages may be different at the start of each charge process, since a series of PDD clock cycles may elapse between the moment transition 1 → 0 is detected during discharge and the moment discharge ends (normally the same number of cycles, *n*, for all discharges). In the case of readings in sensor arrays (where measurements are taken in parallel), this problem is further exacerbated since it is necessary to wait until all the sensors have been read if the charge process is synchronous. Figure 2 shows the effect that this additional time has on the final discharge voltage through two different resistors (the instants for the start of discharge of the two resistors have been superimposed in this figure for clarification). Since these final discharge voltages are the initial ones in the following charge processes, the latter initially show the same difference. The only condition necessary for this difference to appear is that the resistors used for discharge differ from each other. However, this is precisely what happens in all the above calibration methods, since, firstly, *R_C1_* ≠ *R_C2_* must always be met, and, secondly, *R_S_* is usually different from the calibration resistors.

The following section presents a new resistive DIC that can reduce errors in estimating *R_S_*, although the voltages the capacitor is charged at in each cycle differ from each other. This also allows shorter charge times, which in turn decreases *T_E_(R_S_)*.

## 3. Calibration Method for Reducing Error Due to Different Voltages Stored on the Capacitor of a Resistive DIC

**Figure 3:**
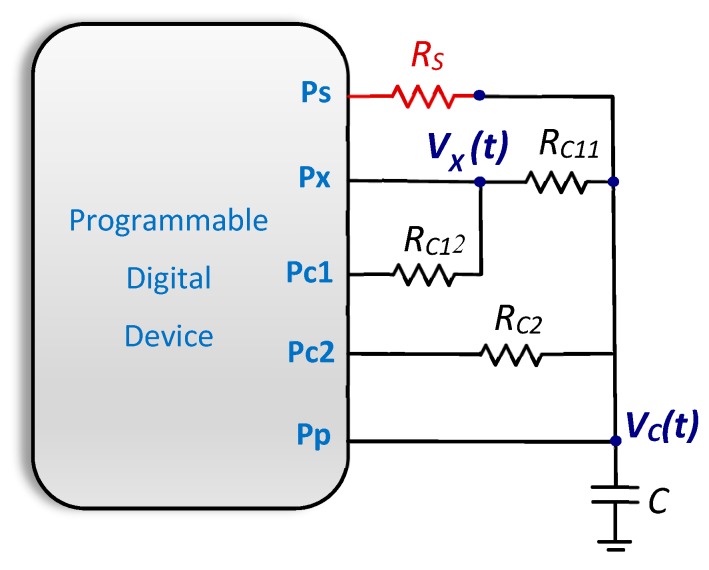
New resistive DIC proposed for error reduction due to different initial voltages stored on the capacitor (resistor/sensor to be estimated shown in red).

To achieve that the initial values of the voltage on the capacitor do not affect the discharge process, we propose a new DIC that is shown in Figure 3. Three calibration resistors are used, *R_C11_*, *R_C12_*, and *R_C2_* apart from the resistor that models the sensor, *R_S_*, and the capacitor, *C*. The sum of the first two resistors will be called *R_C1_*. This sum plays a role equivalent to the first calibration resistor in the TPCM. Moreover, resistor *R_p_*, as used in the DICs of the previous section, is eliminated. The proposed DIC also requires three charge and discharge cycles (three measurement cycles) to estimate *R_S_*. The charge cycles are performed by placing a logic 1 in all the PDD outputs in order to decrease the capacitor charge time, although this will be very similar to that needed if charging through Pp only. All discharges consist of two parts. In the first one, a partial discharge occurs through Pc1 (i.e., through *R_C1_* = *R_C11_* + *R_C12_*) for a preset time *T_A_*. All other pins will be in the HZ state (inputs) during this time. *T_A_* must meet the following relationship

(6) $$T_{X}<T_{A}<T_{RC1}$$

where *T_X_* is the time elapsed from the start of discharge through Pc1 until voltage *V_Th_* is reached in *V_X_*. Since the voltage on the capacitor, at instant *T_X_*, is

(7) $$V_{C}(T_{X})=\left(1+\frac{R_{C11}}{R_{C12}}\right)V_{Th}$$

and, therefore, higher than *V_Th_*; discharge continues through Pc1 up to *T_A_*, without a logic 0 being detected in the Pp pin. The second part of the discharge begins as of *T_A_*, continuing with *R_S_*, *R_C2_*, or *R_C1_* itself, depending on the measurement cycle.

**Figure 4:**
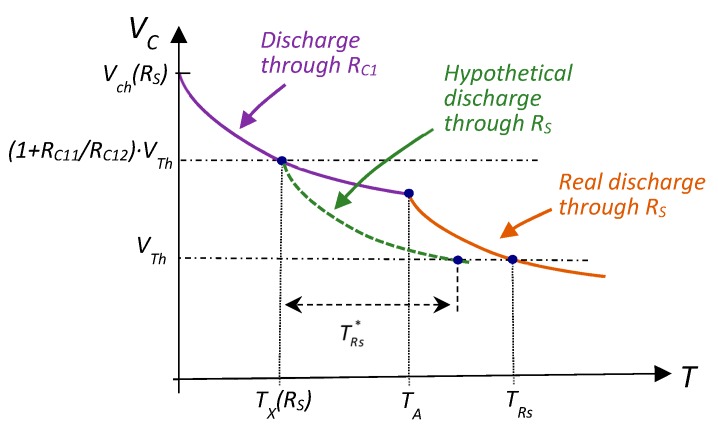
Times taken to estimate *R_S_* in its measurement cycle. Actual discharge through *R_S_* is shown in orange; in green is the length of a hypothetical discharge through *R_S_* if started after a discharge through *R_C1_* up to $T_{X}(R_{S}),\;T_{Rs}^{*}$.

Bearing in mind that the initial discharge voltage may differ in each measurement cycle, it is necessary to distinguish between *V_ch_(R_S_)*, *V_ch_(R_C1_)*, and *V_ch_(R_C2_*), and between *T_X_(R_S_)*, *T_X_(R_C1_)*, and *T_X_(R_C2_)*, where the resistor that the discharge finishes through in a specific measurement cycle is shown in brackets. Moreover, *T_Rs_*, *T_Rc1_*, and *T_Rc2_* maintain their meaning as the total discharge time up to *V_C_ = V_Th_* in each measurement cycle. However, *T_Rs_* is now the sum of the time taken in the first part of the discharge through *R_C1_*, *T_A_*, plus the time taken in the second part of the discharge through *R_S_*. Similarly, *T_Rc2_* is the sum of *T_A_* and discharge time through *R_C2_*. Moreover, *T_Rc1_* is the discharge time through *R_C1_* only, since both the first and second parts of the discharge take place through this resistor. The following variables will be used to set the equation for the new calibration method more straightforwardly: $T_{Rs}^{*}$, $T_{Rc1}^{*}$, and $T_{Rc2}^{*}$. These variables measure the time it would take for the resistor that appears in the sub-index of these variables to discharge *C* from *T_X_* to *V_C_ = V_Th_* in the Pp pin (although these times are not measured in this calibration method). The times defined above, for the case of the *R_S_* measurement cycle, are represented in Figure 4 (a similar figure would be achieved for the *R_C2_* measurement cycle by changing *R_S_* for *R_C2_*).

Only $T_{Rc1}^{*}$ can be found directly, since

(8) $$T_{Rc1}^{*}=T_{Rc1}-T_{X}(R_{C1})$$

However, $T_{R}^{*}$ and $T_{Rc2}^{*}$ can only be found using the equation that describes the *Modified Discharge Process*, as introduced in [13]. This process is similar to the one described above, i.e., a capacitor is discharged through two resistors, *R_i_* and *R_j_* (first through *R_i_* for time *t_i_*, with discharging then ending through *R_j_* for time *t_j_*). Carrying out another capacitor discharge (from the same initial voltage to the same final voltage) through *R_j_* only with discharge time *T_j_* would allow us to know the time *R_i_* would take to carry out the whole discharge, *T_i_*, (without the need to carry out the discharge) by means of the equation (see Appendix A).

(9) $$T_{i}=\frac{T_{j}\cdot t_{i}}{T_{j}-t_{j}}$$

The equivalent equation to this, considering the discharge shown in Figure 4 from instant *T_X_(R_S_)*, is given by

(10) $$T_{Rc1}^{*}=\frac{T_{Rs}^{*}\left(T_{A}-T_{X}(R_{S})\right)}{T_{Rs}^{*}-\left(T_{Rs}-T_{A}\right)}$$

Thus:

(11) $$T_{Rs}^{*}=\frac{T_{Rc1}^{*}\left(T_{Rs}-T_{A}\right)}{T_{Rc1}^{*}-T_{A}+T_{X}(R_{S})}$$

Using this procedure when discharge ends through *R_C2_* finds

(12) $$T_{Rc2}^{*}=\frac{T_{Rc1}^{*}\left(T_{Rc2}-T_{A}\right)}{T_{Rc1}^{*}-T_{A}+T_{X}(R_{C2})}$$

Moreover, times $T_{Rs}^{*}$, $T_{Rc1}^{*}$, and $T_{Rc2}^{*}$ can used to write a modified version of Equation (3) for three discharge cycles that start with the same capacitor voltage as shown in Equation (7), ending in *V_Th_*.

(13) $$R_{S}=\frac{T_{Rs}^{*}-T_{Rc1}^{*}}{T_{Rc2}^{*}-T_{Rc1}^{*}}\left(R_{C2}-R_{C1}\right)+R_{C1}$$

If the results of Equations (8), (11), and (12) are substituted in this expression, *R_S_* can be estimated by the equation

(14) $$R_{S}=\frac{\frac{T_{Rs}-T_{A}}{T_{Rc1}-T_{A}+T_{X}(R_{S})-T_{X}(R_{c1})}-1}{\frac{T_{Rc2}-T_{A}}{T_{Rc1}-T_{A}+T_{X}(R_{c2})-T_{X}(R_{c1})}-1}\left(R_{C2}-R_{C1}\right)+R_{C1}$$

All times are known in this equation, either because they have been measured, *T_Rs_*, *T_Rc1_*, *T_Rc2_*, *T_X_(R_S_)*, *T_X_(R_C1_)*, and *T_X_(R_C2_)*, or because it has been established by the designer, *T_A_*. The set formed by the new DIC proposed in Figure 3 and the new equation for calculating *R_S_* (14) will be called the Short-Time Charging Calibration Method, SCCM.

The basic idea in this new calibration method is to convert a series of initial discharge voltages that can vary between measurement cycles, namely *V_ch_(R_S_)*, *V_ch_(R_C1_)*, and *V_ch_(R_C2_)*, into one indicated by Equation (7) that is constant and independent of the measurement cycle. Equation (14) needs more arithmetic operations and measurements than Equation (3), meaning it therefore has more sources of uncertainty. However, if the variation in the charge voltages of the three measurement cycles is large enough (which is indeed the case when shortening charge times to reduce *T_E_(R_S_)*), the new calibration method may provide better results than TPCM.

## 4. Experimental Results and Discussion

In order to study the proposed DIC and subsequently compare its results with those based on a classic calibration method such as TPCM, both circuits were implemented using a Xilinx FPGA, specifically the Spartan 6 XC6SLX25-3FTG256 model. The working voltage of the I/O blocks of this FPGA, and therefore, the maximum voltage on the capacitor for the discharges, *V_ch_*, was 3.3 V. Output buffers were configured to drive up to 24 mA each. Time-to-digital conversion was performed by a 14-bit counter with a 20 ns time base, which allowed discharge times of up to 327.68 µs. To achieve low measurement uncertainties, we have used the smart capture modules proposed in [28]. FPGA device utilization for the acquisition module was 2% of the Slice LUTs (329), 326 used as logic, and 3 used as route-thrus, and 1% of the Slice Registers (231), 223 used as Flip-Flops, and 8 used as latches. The choice of this device has been mainly due to the versatility of FPGAs. However, it is important to highlight that any PDD is a good candidate for the implementation of the method presented in this article, since no special characteristics of these circuits are required to work with these resistive DICs.

The tests were performed on a set of resistors in the range 270 to 7500 Ω, although the new SCCM allowed values of 10 kΩ to be reached in some of the experiments. As for the calibration resistors, the values chosen for SCCM were *R_C11_* = 557.46 Ω, *R_C12_* = 559.04 Ω, and *R_C2_* = 6165.9 Ω, while for TPCM, they were *R_C1_* = 1116.5 Ω and *R_C2_* = 6165.9 Ω. Note that *R_C1_* for TPCM is the sum of *R_C11_* + *R_C12_* = 1116.5 Ω used in SCCM. A 47 nF capacitor was chosen for *C*. The design rules for this type of DIC are fulfilled with these values, for both the calibration resistors and the capacitor used, as stated in the literature [23,29] (the value of *R_C1_* is around 15% of the range of resistances to be measured and *R_C2_* is around 85%). All the resistors were measured using an Agilent 34401A digital multimeter. In the case of the SCCM method, *T_A_* = 9.68 µs was established for partial discharge time through Pc1, thus complying with the restriction expressed in Equation (6).

In order to compare the results provided for the estimation of *R_S_* using TPCM, as shown in Equation (3), and SCCM, as shown in Equation (14), several tests were carried out for both methods, with varying capacitor charge times. For the comparison, the TPCM has been slightly modified to ensure the capacitor charges as quickly as possible. Therefore, in TPCM, it was decided to charge through all the available pins, as in SCCM. Furthermore, as resistor *Rp* in Figure 1a slows down the charge process, it was decided to replace it with a short circuit.

A 12-bit counter was implemented in the FPGA, *b_11_b_10_...b_1_b_0_*, to control the charge time in both methods. These bits will determine charge time. In order to simplify the hardware design in the FPGA, bearing in mind that a range of charge times must be implemented, the most significant output bit of this counter, *b_11_*, was used to control maximum charge time, as indicated below. When the charge process starts, the counter resets, and the count starts. Charging continues as the counter advances, as long as *b_11_* remains 0. The charge process (and the counter advance) ends at the moment *b_11_* becomes 1, with the discharge process then starting. Therefore, the maximum charge time is 2^11^·*T_CK_*, where *T_CK_* = 20 ns is the period of the clock signal used in the counter. In our case, this maximum charge time is therefore 40.96 µs. Other counter output bits were used to find shorter charge times. For example, *b_10_* was used as the control bit to find a charge time of 20.48 µs. Bit pairs were used to make this control more precise when charge times were shorter. Therefore, the charge time was 1.92 µs if the charge stops the first time bits *b_6_* and *b_5_* are simultaneously 1. The charge times used for the comparison between the two calibration methods were 1.92 µs, 2.56 µs, 3.84 µs, 5.12 µs, 10.24 µs, 20.48 µs, and 40.96 µs. The figure of merit to be used for the comparison will be the maximum relative error found for the set of 500 estimations made for each resistor with each charge time.

**Figure 5:**
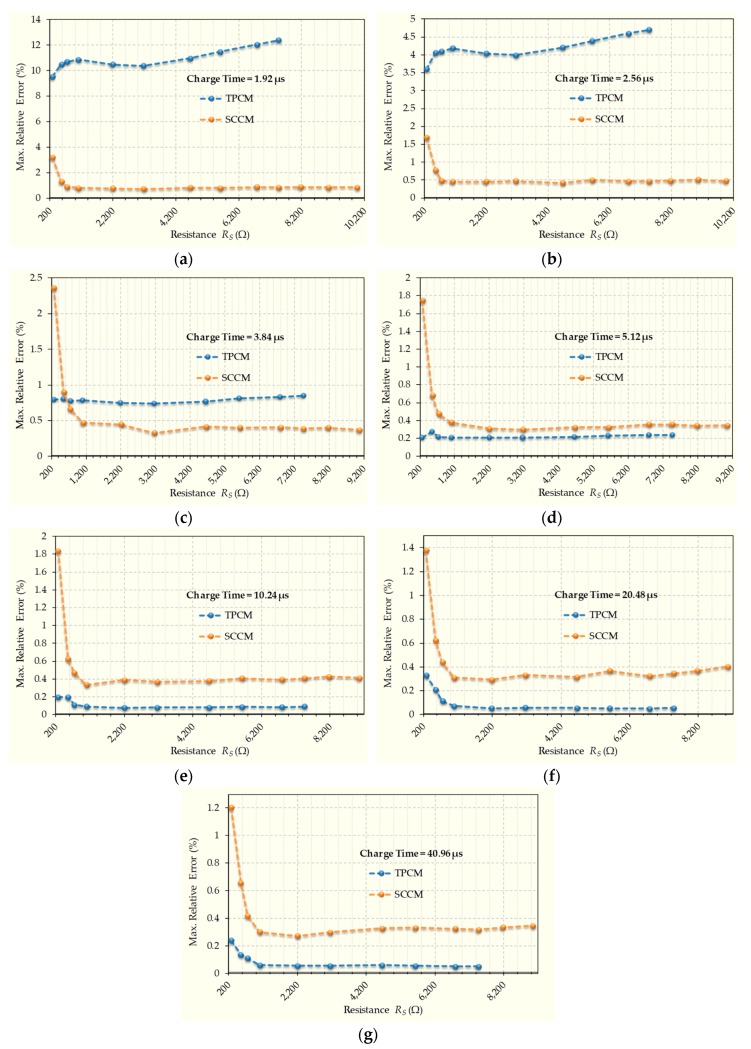
Comparison of maximum relative errors in estimating *R_S_* when using the Short-Time Charging Calibration Method (SCCM) or TPCM. Each graph represents a different capacitor charge time. (**a**) charge time of 1.92 µs (**b**) charge time of 2.56 µs, (**c**) charge time of 3.84 µs, (**d**) charge time of 5.12 µs, (**e**) charge time of 10.24 µs, (**f**) charge time of 20.48 µs, (**g**) charge time of 40.96 µs.

The maximum relative errors found in the estimation of *R_S_* with the charge times indicated above are shown in Figure 5 for both calibration methods. The results for the shortest charge time are shown in Figure 5a, with charge time increasing in the different figures up to Figure 5g, which shows the results for the longest charge time. Figure 5 shows two main features. Firstly, the maximum relative error with SCCM hardly varies between the different charge times. Indeed, for most resistors, this error varies between 0.5% and 0.3% and, only for very low resistance values, this error is higher with small charge times. This increase in the error for small resistances is caused by the quantization errors, since, in the case of SCCM, these errors are more severe because the time measurements of *T_X_(R_S_)*, *T_X_(R_C1_),* and *T_X_(R_C2_)* are smaller than *T_Rs_*, *T_Rc1_*, or *T_Rc2_*. Obviously, this can be compensated by an increase in the clock frequency and/or the capacitance, *C*. However, these changes must be carefully evaluated considering the drawbacks that they entail (higher power consumption, increasing noise, and *T_E_(R_S_)*). Another more interesting way that could be used to achieve the reduction of quantization errors would be to use the method proposed in [16] together with the SCCM. However, this is beyond the objectives of this article.

Secondly, as expected, the maximum relative error in TPCM is large for small charge times, with a maximum of 12.4% for a charge time of 1.92 µs and a resistance value of 7464 Ω. As the charge time increases, this error decreases for all resistance values in the range, until values slightly below 0.1% are reached for high resistances with a maximum charge time of 40.96 µs. SCCM almost always outperforms TPCM in accuracy for short charge times up to 3.84 µs. The exceptions to this are resistances of less than 600 Ω with a charge time of exactly 3.84 µs. Relative errors in both methods are very similar for a charge time of 5.12 µs. For higher charge times, TPCM slightly outperforms SCCM. However, even in these cases, the difference in the maximum relative errors is only 0.2% for almost the entire resistance range. Therefore, SCCM makes it possible to reduce the charge time more than 20-fold with a very moderate increase in error. Secondly, it is observed that SCCM allows higher resistance values to be estimated than in the case of TPCM. This is because part of the discharge through *R_S_* is via *R_C1_*, meaning that the total discharge time for *R_S_* > *R_C1_* is shortened and discharges for higher resistance values can be measured with the designed counter.

The reason why error with high charge times is greater in SCCM than in TPCM is related to uncertainty in the measurement of *T_X_*, *u(T_X_)*, (independent of the measurement cycle, because to find *T_X_*, the discharges are always through *R_C1_*). Indeed [30] shows that uncertainty in measuring discharge time through any *R*, *u(T_R_)*, depends proportionally on the circuit’s electrical noise in the measurement node (in this case, the capacitor node), $\varepsilon_{C}$, and inversely on the absolute value of the derivative of the discharge curve (1) with respect to time at the measured instant.

(15) $$u(T_{R})\propto \varepsilon_{C}\cdot \left(R+R_{o}\right)\cdot C\approx \varepsilon_{C}\cdot R\cdot C$$

Finally, as the *V_X_(t)* discharge curve in Figure 3 is given by

(16) $$V_{X}(t)=\frac{R_{C12}}{R_{C1}}V_{ch}\cdot e^{\frac{-t}{\left(R_{C1}+R_{o}\right)C}}$$

it is easy to find that

(17) $$u(T_{X})\propto \varepsilon_{X}\cdot \left(R_{C1}+R_{o}\right)\cdot C\approx \varepsilon_{X}\cdot R_{C1}\cdot C$$

where $\varepsilon_{X}$ is the electronic noise of node *X* in Figure 3. The relationship between *u(T_Rc1_)* and *u(T_X_)* is therefore found by adapting Equation (15) and using Equation (17).

(18) $$\frac{u(T_{X})}{u(T_{Rc1})}\propto \frac{\varepsilon_{X}}{\varepsilon_{C}}$$

**Figure 6:**
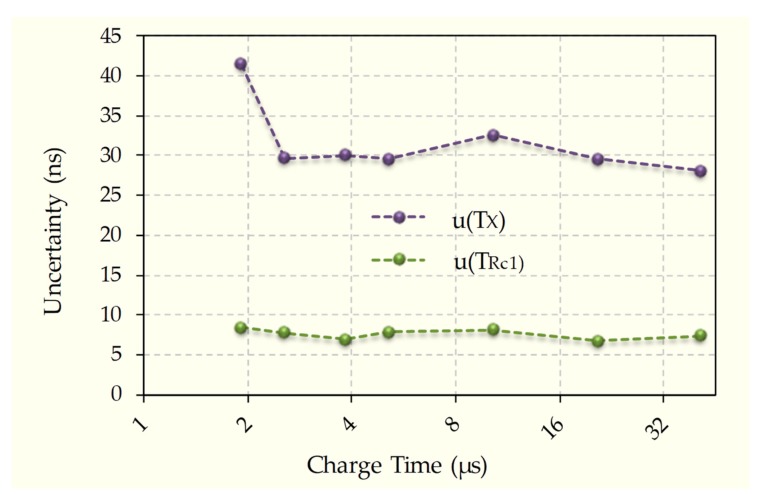
Comparison of uncertainties in discharge times measured in the capacitor node and in node *X* when discharge occurs through *R_C1_*. Uncertainty in the capacitor node in Figure 3, *u(T_Rc1_)*, is shown in green, while uncertainty in the *X* node, *u(T_X_)*, is shown in purple, both for different charge times (in log2 scale).

However, the electronic noise in node *X* is higher than that in the capacitor node, which is mainly due to the fact that the capacitor itself filters this noise. Therefore, the ratio in Equation (18) should be greater than one and approximately constant. Furthermore, the relationship between uncertainties will vary for each implementation of a single DIC. Figure 6 shows the results for *u(T_Rc1_)* and *u(T_X_)* in our implementation of the circuit for the different charge times. Except for a small increase in *u(T_X_)* when the charge time is 1.92 µs, the uncertainties are approximately constant, since the resistance carrying out the capacitor discharge is the same in all cases, *R_C1_*, and the relationship between these uncertainties is $u(T_{X})\approx 4\cdot u(T_{Rc1})$. The observed growth in uncertainty for a charge time of 1.92 µs is due to the final charging voltage already being much less than *V_DD_*, and therefore *T_X_* is a smaller value that is more affected by quantization errors. This high value of *u(T_X_)* compared to *u(T_Rc1_)* is responsible for the maximum relative error of SCCM being higher than that of TPCM for longer charge times, especially considering that there are three times to be measured in node *X*, each with their corresponding uncertainties, as shown in Equation (14).

**Figure 7:**
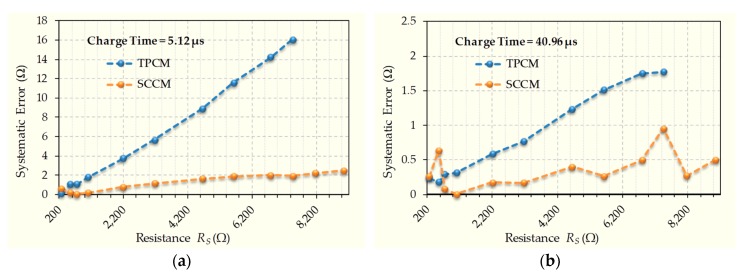
Systematic error for TPCM and SCCM for various charge times. (**a**) Charge Time of 5.12 µs. (**b**) Charge Time of 40.96 µs.

In order to compare the results for SCCM with those for TPCM, minimizing the influence of any uncertainty in the estimation of *R_S_*, the average of the 500 estimations of *R_S_* for each charge time has been calculated, and this value has been taken as the systematic estimation of each method. We call the absolute difference between the real value of *R_S_* and this systematic estimation systematic error. Figure 7 shows the difference between these systematic errors for both methods in the case of a charge time close to the minimum, 5.12 µs, as shown in Figure 7a, and, for the maximum charge time, 40.96 µs, as shown in Figure 7b. In Figure 7b, the systematic error is lower in SCCM (except for resistance value 556.4 Ω), unlike the relative errors shown in Figure 5g. This shows that the main cause of errors in SCCM is the higher uncertainty of the different *T_X_* measurements. The form of systematic errors in TPCM is typical, increasing with resistance value [30]. However, systematic errors increase more slowly in SCCM, meaning that the advantage for SCCM increases as the resistance value increases.

## 5. Conclusions

Direct Interface Circuits are a simple way to read a resistive sensor in digital form without the need for analog-to-digital converters. The simplest versions of these circuits need only a few passive elements (resistors and a capacitor) to provide this information, *R_S_*. Even with these simple circuits, the accuracy achieved in measurement is quite high, with errors of just a few tenths of a percent. However, as in any circuit, they also have a series of limitations, most notably the time needed to carry out the estimation of *R_S_*, *T_E_(R_S_)*. Since this time is the sum of various charge and discharge times, reducing each of them helps decrease *T_E_(R_S_)*. 

Accuracy in the estimations when using a DIC is only possible if the final voltages stored on the capacitor are the same in the different charge cycles, which, since the initial voltages that charging starts from differ from each other, can only be achieved if capacitor charge times are long enough. This slows down the operation of the DIC, which can be a serious problem when multiple resistive sensors need to be read in a system.

This article presents a method to reduce charge times that consists of a new DIC architecture (in which only passive components are still used) together with a new calculation method for the estimation of *R_S_*, which we call the Short-Time Charging Calibration Method, SCCM. The method is based on performing two measurements within a single discharge process, while a classic DIC only performs one. These new measurements only require an additional pin for the programmable digital device used in the DIC, and the information they provide means that the initial voltages in the discharge processes do not necessarily have to be the same in order to correctly estimate *R_S_*. For charge times of 1.92 µs, this method has achieved errors very similar to those in a classic DIC with charge times of 40.96 µs, the difference being around 0.2%–0.3% for almost all resistance values in the tested range. Furthermore, SCCM can measure higher resistance values for the same maximum discharge time.

## Appendix A

If *t_i_*, *t_j_*, and *T_j_* are the times defined in Section 3, then it is possible to find *T_i_* using Equation (9). To demonstrate this, *t_i_*, *t_j_*, and *T_j_* could be written as:

(A1) $$t_{i}=(R_{i}+R_{o})C\ln\left(\frac{V_{ch}}{V_{X}}\right)$$

(A2) $$t_{j}=(R_{j}+R_{o})C\ln\left(\frac{V_{X}}{V_{Th}}\right)$$

(A3) $$T_{j}=(R_{j}+R_{o})C\ln\left(\frac{V_{ch}}{V_{Th}}\right)$$

where *V_X_* is the final voltage reached during the partial discharging through *R_i_*. With these equations, we can write:

(A4) $$\begin{array}{c} \frac{T_{j}\cdot t_{i}}{T_{j}-t_{j}}=\frac{(R_{j}+R_{o})C\ln\left(\frac{V_{ch}}{V_{Th}}\right)\cdot (R_{i}+R_{o})C\ln\left(\frac{V_{ch}}{V_{X}}\right)}{(R_{j}+R_{o})C\ln\left(\frac{V_{ch}}{V_{Th}}\right)-(R_{j}+R_{o})C\ln\left(\frac{V_{X}}{V_{Th}}\right)}=\\ =\frac{(R_{i}+R_{o})C\ln\left(\frac{V_{ch}}{V_{Th}}\right)\ln\left(\frac{V_{ch}}{V_{X}}\right)}{\ln\left(\frac{V_{ch}}{V_{Th}}\right)-\ln\left(\frac{V_{X}}{V_{Th}}\right)}=(R_{i}+R_{o})C\ln\left(\frac{V_{ch}}{V_{Th}}\right)=T_{i}\;□ \end{array}$$
